# Deficiency of Adenosine Deaminase 2 (DADA2): Updates on the Phenotype, Genetics, Pathogenesis, and Treatment

**DOI:** 10.1007/s10875-018-0525-8

**Published:** 2018-06-27

**Authors:** Isabelle Meyts, Ivona Aksentijevich

**Affiliations:** 1 Department of Pediatrics, Department of Microbiology and Immunology, University Hospitals Leuven, Leuven, Belgium; 2 Inflammatory Disease Section, National Human Genome Research Institute, Bethesda, USA

**Keywords:** Adenosine deaminase 2, DADA2, vasculitis, polyarteritis nodosa (PAN), stroke, pure red cell aplasia (PRCA), immune thrombocytopenia, neutropenia, immunodeficiency

## Abstract

Deficiency of ADA2 (DADA2) is the first molecularly described monogenic vasculitis syndrome. DADA2 is caused by biallelic hypomorphic mutations in the *ADA2* gene that encodes the adenosine deaminase 2 (ADA2) protein. Over 60 disease-associated mutations have been identified in all domains of ADA2 affecting the catalytic activity, protein dimerization, and secretion. Vasculopathy ranging from livedo reticularis to polyarteritis nodosa (PAN) and life-threatening ischemic and/or hemorrhagic stroke dominate the clinical features of DADA2. Vasculitis and inflammation can affect many organs, explaining the intestinal, hepatological, and renal manifestations. DADA2 should be primarily considered in patients with early-onset fevers, rashes, and strokes even in the absence of positive family history. Hematological manifestations include most commonly hypogammaglobulinemia, although pure red cell aplasia (PRCA), immune thrombocytopenia, and neutropenia have been increasingly reported. Thus, DADA2 may unify a variety of syndromes previously not thought to be related. The first-line treatment consists of TNF-inhibitors and is effective in controlling inflammation and in preserving vascular integrity. Hematopoietic stem cell transplantation (HSCT) has been successful in a group of patients presenting with hematological manifestations. ADA2 is highly expressed in myeloid cells and plays a role in the differentiation of macrophages; however, its function is still largely undetermined. Deficiency of ADA2 has been linked to an imbalance in differentiation of monocytes towards proinflammatory M1 macrophages. Future research on the function of ADA2 and on the pathophysiology of DADA2 will improve our understanding of the condition and promote early diagnosis and targeted treatment.

## Introduction

Deficiency of adenosine deaminase type 2 is an autosomal recessive disease resulting from loss-of-function (LOF) mutations in *ADA2*, formerly named *CECR*1 (*cat eye syndrome chromosome region*, *candidate* 1) gene [1, 2]. Initially recognized as a syndrome that manifests with fevers, polyarteritis nodosa, livedo racemosa, early-onset stroke, and mild immunodeficiency, the clinical phenotype has expanded significantly since it was first described in 2014 [1, 2]. Clinical presentation and age of onset vary widely even among related patients, and the most severe manifestations include marrow aplasia, PRCA, neutropenia, liver disease, and neurological impairments.

Adenosine deaminase 2 (ADA2) was first described as the residual source of adenosine deaminase activity in the spleen of a patient with severe combined immunodeficiency (SCID) due to adenosine deaminase deficiency (ADA; also known as ADA1) [3]. Adenosine deaminase proteins regulate purine metabolism by breaking down adenosine (Ado) and 2′-deoxyadenosine (dAdo) inside cells. In the absence of ADA1, toxic deoxyadenosine nucleotides accumulate in lymphocytes, ultimately leading to T-B-NK-SCID phenotype [4]. ADA1 has significantly higher affinity for its substrates Ado and dAdo than ADA2. The two proteins are partially homologous; however, they have distinct structure and possibly diverse functions (Table 1) [3]. Preliminary studies suggest that in addition to its deaminase activity, ADA2 may have a growth factor activity [5, 6]. Moreover, patients with ADA2 deficiency do not accumulate deoxyadenosine nucleotides and have normal ADA1 activity [1, 2, 7].

**Table 1:** Characteristics of ADA1 and ADA2

	ADA1	ADA2
Gene	*ADA*	*ADA2*
Chromosome	20q13.12	22q11.1
Expression	Ubiquitously, lymphocytes, erythrocytes	Myeloid cells, lymphocytes, lung, BM, spleen, thymus
Protein structure*	41-kDa monomer, binds to cell surfaces via CD26	59-kDa monomer-homodimer, glycosylated, binds to heparin/glycosaminoglycan/cell surface receptors
Cellular localization	Intracellular	Secreted, lysosomal?
Function	Adenosine deaminase	Adenosine deaminase, regulation of cell proliferation and differentiation
Optimum pH ADA function	7.5	6.9
Inhibited by EHNA^#^	Yes	No
Clinical phenotype when deficient	T-B-NK-SCID	Deficiency of ADA2 (DADA2) early-onset polyarteritis nodosa (PAN)
Other references		Cat eye syndrome, ADGF subfamily

^#^*EHNA* erythro-9-(2-hydroxy-3-nonyl)adenine

*Based on the GeneCards database

Without treatment, SCID due to ADA1 deficiency is fatal early in life. Hematopoietic stem cell transplantation (HSCT) is the major treatment for SCID-ADA; other treatment modalities include enzymatic replacement therapy (ERT) and gene therapy [8]. Relative to SCID-ADA, DADA2 has a milder phenotype notwithstanding the report of patients who died in early childhood [2, 9]. Interestingly, the absence of one of the enzymes is not compensated for by the other enzyme. This suggests that ADA1 and ADA2 have non-redundant functions. Here, we review recent advances in understanding the pathophysiology, clinical presentation, and treatment of patients with DADA2. In 2014, two independent groups, Zhou et al. and Navon-Elkan et al., described the first 34 patients with disease-associated mutations in ADA2. During the past 3 years, over 150 new patients of many ancestries have been reported in the literature. Given the allele frequency of pathogenic *ADA2* variants, ADA2 deficiency may be more common than anticipated, in particular in specific populations. Better understanding of the full clinical spectrum of DADA2 and the availability of molecular and biochemical diagnostics will ultimately lead to improvements in diagnosis, management, and clinical outcome of these patients.

## Adenosine Deaminase Function

**Fig. 1:**
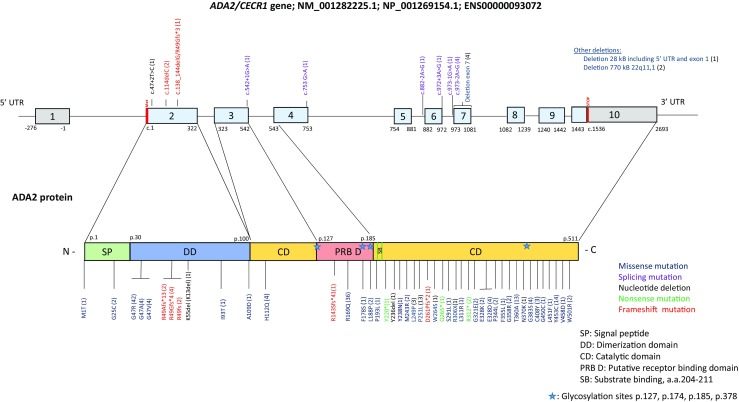
Schematic representation of disease-associated mutations in the *ADA2* gene and ADA2 protein

Although ADA2 has long been regarded as an isozyme of ADA1, they differ in structure, cellular localization, and expression (Table 1). ADA1 is a 41-kDa monomer protein that is present in all human tissues and with highest expression in T and B lymphocytes. ADA1 has a critical function in adaptive immune system development although the mechanism is as yet unclear [10–12]. ADA2 is a 59-kDa protein that forms homodimers and is secreted into the extracellular space [6, 13]. ADA2 is highly expressed in myeloid cells and produced by activated monocytes, macrophages, and dendritic cells [5, 14]. The crystal structure of human ADA2 revealed large differences in the arrangement of the substrate-binding pockets, explaining the difference in catalytic parameters of ADA1 and ADA2 and their specificity for inhibitors [15]. ADA2 has a 100-fold higher Michaelis Konstant for adenosine (*K*_m_ = 2 mM) than ADA1, which is a supraphysiologic concentration—the normal adenosine concentration in plasma and extracellular fluids being 0.2 μM—and this at an acidic pH of 6.9 [3]*.* That means that the rate of adenosine deamination catalyzed by ADA2 is close to zero at the physiological adenosine concentrations. ADA2 displays lower sensitivity to many ADA1-specific inhibitors. Erythro-9-(2-hydroxy-3-nonyl)adenine (EHNA) is the only known inhibitor that binds to ADA1 and not to ADA2 and as such is used to discriminate ADA1 vs. ADA2 activity in biochemical assays [3]. ADA2 has a longer sequence than ADA1 with additional domains including an N-terminal domain that is likely responsible for its growth factor activity and a putative receptor-binding domain (PBR) [15]. The cytokine-like growth activity of ADA2 requires binding to yet unidentified cell surface receptors. Interestingly, unlike ADA1, ADA2 does not associate with the cell surface glycoprotein CD26 which serves as a binding protein for extracellular ADA1 in humans, aiding in reducing local adenosine levels [16, 17]. The crystal structure analysis of ADA2 also identified several glycosylation sites that are common in eukaryotic-secreted proteins (Fig. 1) [15].

ADA2 shares high sequence similarity with adenosine deaminase growth factors (ADGFs). The founding member of ADAGFs was identified from media of insects (flesh flies; *S. peregrina*), and its deaminase activity was found to be essential to their growth [18–21]. Deletion of the major ADGF-A in *Drosophila* causes larval death, and this phenotype was rescued by expression of ADGF-A in hemocytes (blood cells) alone [22]. Although ADA2 is found in most species, mice lack an *ADA2* ortholog and that has hampered in vivo studies of ADA2 function. Partial triplication of the 22q11.2 chromosome containing the *ADA2* gene in humans results in developmental abnormalities [23]. The knockout of ADGF/ADA2 homologues in frogs causes developmental abnormalities, while transgenic expression of ADA2 in mice results in abnormal development of heart and kidney [24, 25]. Together, these data suggest that ADA2 has a role in the regulation of cell proliferation and differentiation independent of its catalytic activity. The possible role of ADA2 in bone marrow differentiation remains to be investigated.

Myeloid cells are known to release ADA2 during inflammatory responses, and high levels of ADA2 are found in plasma samples and pleural effusions of patients with infectious diseases or chronic inflammation. ADA2 activity is highly elevated in patients with Crohn’s disease, autoimmune diseases, chronic hepatitis or cirrhosis, AIDS, and tuberculosis [14, 26–32]. ADA2 activity appears to correlate with disease activity and relapse. As adenosine signaling must be tightly regulated, ADA2 may have a role in the degradation of extracellular adenosine derived from excessive ATP breakdown at the site of inflammation. However, given the enzymatic characteristics of ADA1 and ADA2 as explained above, it is likely that in inflamed tissues, both ADA1 and ADA2 may be needed to control the levels of extracellular adenosine. Although ADA1 does not have any signal sequences required for secreted protein significant amounts of ADA1 are found to circulate in plasma. In all, ADA1 plays the lead role in deamination of both intracellular and extracellular adenosine, which is supported by the finding of increased adenosine and deoxyadenosine in patients with the SCID due to ADA [1] deficiency [33].

Both ADA1 and ADA2 bind to proteoglycans and possibly adenosine receptors on immune cells, which suggest their role in cell activation and signaling [34, 35]. ADA1 and ADA2 bind to distinct types of myeloid cells and lymphocytes, with ADA2 preferentially binding to neutrophils, CD16+ monocytes, B cells, and NK cells [36]. In vitro data suggested that ADA2 binds to a receptor on the cell surface of T cells to induce T cell-dependent differentiation of monocytes into macrophages [5]. This data is of particular interest in view of the recent identification of immune dysregulation in DADA2 patients.

## Genetics of DADA2

DADA2-associated mutations are located over the entire coding region of *ADA2* (Fig. 1). *ADA2* is a highly polymorphic gene that harbors over 300 missense substitutions and indels, which requires careful evaluation of candidate causal variants. There is also an excess of copy number variants (CNVs) across the gene locus (http://exac.broadinstitute.org) (http://gnomad.broadinstitute.org). One family with two affected siblings was found to carry a homozygous 770-kb genomic deletion that includes *IL-17RA* and a number of other genes in addition to *ADA2* [37]*.* DADA2-associated mutations are either novel (unreported) or found at a low allele frequency (< 0.001) in public databases, consistent with recessive inheritance of the rare disease. Up until the time of writing, 61 disease-causing mutations have been described and most of them are missense variants, although genomic deletions, nonsense, and splicing mutations have also been reported [1, 2, 7, 9, 37–60]. The clinical significance of novel candidate variants cannot be inferred from their allele frequency alone [61]. Parental samples should be genotyped to demonstrate a proper inheritance pattern, and their impact on protein function needs to be assessed by in silico analysis and/or a biochemical assay. In a small number of patients, DNA sequencing revealed only one demonstrable pathogenic mutation despite low or absent ADA2 activity [50]. These patients need to be investigated for the presence of structural or non-coding causal variants, which requires more sophisticated analyses that are typically beyond the scope of routine genetic testing. Siblings of DADA2 patients should be counseled and encouraged to consider genetic testing. Importantly, some carriers of pathogenic *ADA2* mutations with ADA2 activity levels in the carrier range exhibit mild and/or late-onset features of the disease. These findings are at present unexplained but a gene dosage effect in the presence of modifier genes cannot be excluded. From a clinical point of view, in a patient with a suspect clinical phenotype, one might consider first to test the plasma ADA2 activity, e.g., by high-performance liquid chromatography or ELISA assay as this is the proof of ADA2 deficiency. Thereafter, DNA sequencing of ADA2 can be performed, and in patients with a single identified pathogenic variant, it should be complemented with microarray, quantitative polymerase chain reaction, and/or whole genome sequencing (in research setting). In the presence of confirmatory protein ADA2 assay, search for a second mutation is more relevant for genetic counseling of other family members, e.g., siblings. Turnaround time both for plasma activity as well as traditional Sanger sequencing is very short (down to 1 week). If the ADA2 plasma activity and level proves normal, gene panel or exome sequencing should be considered to pin down an alternative molecular diagnosis.

The majority of patients with DADA2 are compound heterozygous for missense mutations. The most common disease variants are p.Gly47Arg (p.G47R), p.Gly47Ala (p.G47A), p.Arg169Gln (p.R169Q), and p.Tyr453Cys (p.Y453C) [1, 2]. These mutations are found in patients of different ancestries; however, some of them are more common in founder populations. The homozygous p.G47R mutation has been identified in all patients of Georgian-Jewish and most Turkish patients with early-onset PAN [2]. The estimated carrier frequency of p.G47R mutation is 10% in the Georgian-Jewish population—somewhat lower (1:500) in the Turkish population—while this variant is unreported in the European population. The p.R169Q variant is a founder mutation in the Dutch, Belgium, and Finnish populations, while p.Thr360Ala is more common in Italian patients [38, 50, 57]. Haplotype analysis in Dutch patients suggested an ancient founder based on the identification of a small 50-kb common haplotype carrying p.R169Q [38]. The carrier frequency is about 1:500 in Northern European populations, significantly lower in African and Latino populations, and absent in Asian populations. Although DADA2 patients have been identified in many populations, the disease is likely underdiagnosed.

## Genotype-Phenotype Correlations

The published case series of DADA2 patients revealed a large phenotypic variability that cannot be fully explained by the impact of causal mutations on protein function and a degree of residual enzymatic activity. Patients homozygous for the same founder mutation may have variable age of presentation, frequency, and intensity of symptoms [38]. In a large family of Iraqi descent, four adult family members with homozygous mutations were symptom-free [49]. This implies a role for other genetic and epigenetic modifiers, and possibly environmental factors in the disease expressivity. For instance, Trotta et al. described onset of the inflammatory phenotype and vascular flares following proven bacterial infection [57].

Parents of DADA2 patients are typically unaffected which means that 50% of normal enzymatic activity is sufficient for the protein functions. Preliminary reports suggest that patients who have undetectable ADA2 activity tend to have more severe phenotype, although larger studies are needed to confirm this observation [38]. Whether heterozygosity for *ADA2* mutation predispose to late-onset stroke, polygenic vasculitis, and other cardiovascular disease remains to be investigated. Genome-wide association studies (GWAS) did not link common variants in this gene locus to vascular disorders [62, 63].

Pathogenic ADA2 mutations have been linked to other previously described phenotypes. ADA2 deficiency may account for some patients with Sneddon syndrome, a disease characterized by livedo racemosa, leg ulcerations, intermittent fever, ischemic stroke, and antiphospholipid antibodies, typically manifesting in adulthood and more commonly occurring in women [39]. Homozygous p.G47R mutation has been reported in a Jewish patient diagnosed with HHV-8-negative Castelman’s disease [40].

## Pathogenesis of Deficiency of ADA2

Deficiency of ADA2 is associated with monocyte-macrophage polarization towards the M1 subset, and M1 macrophages are known to promote inflammation and tissue damage. Increased production of proinflammatory cytokines was found in skin biopsies and blood samples of DADA2 patients [1, 7, 36]. ADA2-deficient patient monocytes and U937 cells induced disruption of the cell junctions in cocultured monolayers of human microvascular endothelial cells [1]. Thus, ADA2 appears to be critical for the maintenance of vascular integrity. This has been reiterated in an in vitro 3D model by Zhu et al., which showed that *ADA2* in M2-like glioma-associated macrophages mediate cross talk between macrophages and pericytes via a platelet-derived growth factor-dependent pathway resulting in neo-angiogenesis [64].

The vascular phenotype of DADA2 was observed in zebrafish embryos injected with *cecr1* morpholinos, which lead to a significant increase in intracranial bleeding. In addition, the disruption of *cecr1* expression in *mpx*-GFP transgenic fish caused a marked reduction in neutrophil numbers [1].

Recently, several studies demonstrated a type I interferon gene expression signature in patients with DADA2 despite the lack of intracranial calcifications, which is the hallmark of Aicardi-Goutieres syndrome. The molecular basis of type I IFN signature has not been investigated [41, 46, 51].

## Clinical Presentation

The clinical presentation in patients with DADA2 has many faces. At present time, 161 patients have been reported in the literature: 47% female, 53% male [7, 9, 37–39, 41–60, 65–70]. The highly variable clinical presentation renders early diagnosis difficult. Onset of disease is usually in childhood with 24% of reported patients presenting before 1 year of age, 77% presented before the age 10 years. However, adult onset has also been described with the oldest patient presenting at age 59 years with leg ulceration. Mortality is significant with up to 8% of patients succumbing to the disease before the age of 30 years; cause of death includes complications of recurrent stroke or infection.

### Vasculitis/Vasculopathy

**Fig. 2:**
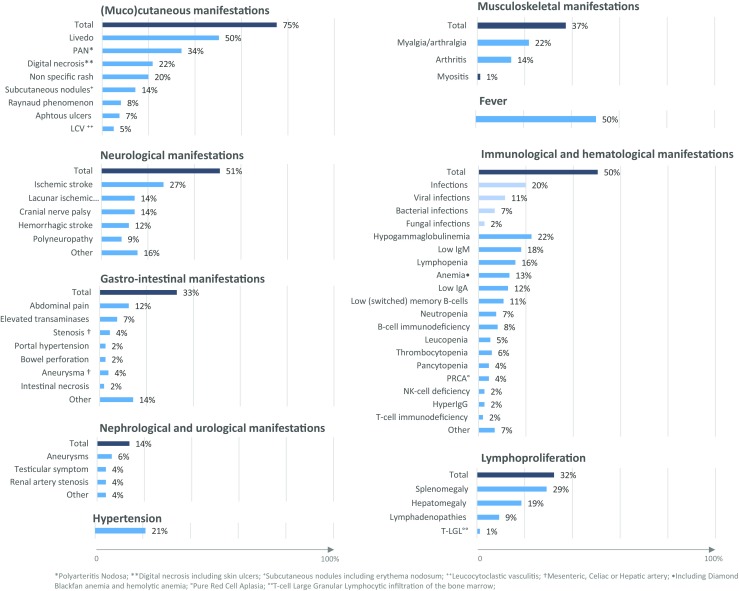
Bar diagram representing the percentage of reported DADA2 patients in whom a given phenotype has been reported

Vasculopathy of small- and medium-sized arteries is the major clinical feature of DADA2 (Fig. 2). Skin and central nervous system are most commonly involved yet other tissues are affected to various degrees (gastrointestinal, liver, renal, coronary). Fever with increased erythrocyte sedimentation rate or CRP was reported in 50% of patients. Arthralgia, myalgia, and arthritis mainly affecting small joints are noted in a minority of patients.

Cutaneous manifestations are the most common feature of DADA2 and are found in > 75% of patients (Fig. 2). Skin biopsy of livedoid rash revealed a predominant interstitial neutrophil and macrophage infiltration with perivascular T lymphocytes and without overt vasculitis, at least in younger patents [1]. The skin biopsy of patients with cutaneous PAN showed non-granulomatous, necrotizing arteriitis of small- or medium-sized muscular arteries [1, 2]. Other types of non-specific skin rash such as nodular rash and macular erythema have been also described.

Of all reported patients, 50% have experienced one or more neurological events. Typical MRI image consists of acute or chronic lacunar ischemic infarcts located in the deep-brain nuclei and/or the brain stem and sparing the subcortical white matter. Onset of ischemic strokes can be as early as 5 months of age. Hemorrhagic stroke and intracranial bleeding seem to be part of the clinical spectrum, even though the initial descriptions were blurred by the concomitant use of aspirin, antiplatelet agents, or warfarin [1, 2]. Importantly, there may be significant underestimation of the cerebral involvement as transient ischemic attacks affecting one or more cranial nerves, large vessel involvement, and a cerebral vessel aneurysm have also been described [1, 2]. Other rare manifestations include spastic diplegia or paraplegia, peripheral polyneuropathy (with perineuritis), ataxia, neurosensory hearing loss, mononeuritis multiplex, labyrinthitis, encephalopathy, and cerebral atrophy [2, 38, 42, 44, 46, 48, 49].

The vasculitis can also affect the liver, kidney, and other organs (Fig. 2). Several case series reported arterial hypertension, renal vessel artery aneurysm, renal artery stenosis, kidney inflammation with dense lymphocytic infiltration, and glomerular scarring [1, 2, 49]. GI manifestations include abdominal pain and inflammatory bowel disease in up to 10% of patients [1, 41, 44, 45, 66, 67]. Pathology report showed a CVID-like inflammatory bowel phenotype with absent plasma cells [7]. Intestinal necrosis, bowel perforation, stenosis, or aneurysm of the mesenteric artery/celiac artery are noted in rare cases [1, 2, 41, 50]. Liver disease is another important feature of DADA2. Elevated transaminases, hepatosplenomegaly, and portal hypertension are observed in < 10% of patients [1, 44]. Histopathology analyses of the liver showed nodular regenerative hyperplasia and/or hepatoportal sclerosis that could potentially lead to end-stage liver disease [1].

### Immunodeficiency

In the initial reports on DADA2, the inflammatory phenotype was predominant. Nevertheless, Zhou et al. reported hypogammaglobulinemia with consistently low IgM levels in a subset of patients [1]. They also noted decreased number of memory B cells in the peripheral blood and a modest reduction in terminal differentiation of B cells and immunoglobulin-secreting cells following stimulation with CD40L and IL-21. A bone marrow analysis of the B cell compartment in one patient showed normal B cell maturation yet reduced numbers of CD138+ plasma cells [1]. Subsequently, Schepp et al. identified nine patients with DADA2 in their cohort of patients presenting with common variable immunodeficiency (CVID) and onset of immunodeficiency prior to age 10 years [45, 65]. These patients presented with recurrent sinopulmonary infections typical of humoral immunodeficiency but also experienced increased susceptibility to herpes virus infections. Systematic B cell immunophenotyping was not performed. At present time, 25% of patients have been described to have hypogammaglobulinemia [1, 7, 38, 41, 43, 45, 49, 50, 57, 67], while lymphopenia is reported in 15% of patients. B cell lymphopenia and low switched memory B cells are found in 10%. The pathophysiology of the immunodeficiency in DADA2 is unclear. The leading hypothesis is that the inflammatory status inhibits B cell differentiation and function. In line with this hypothesis, Schepp et al. showed that treatment with etanercept resulted in increasing serum IgM in one patient [45].

Lymphoproliferation is another important feature of DADA2. Typically, patients present with generalized lymphadenopathy (> 10%) and splenomegaly (up to 30%) [1, 7, 37, 38, 42, 45, 47, 50, 55–57, 60]. Recently, patients with a phenotype resembling autoimmune lymphoproliferative syndrome (ALPS) have been reported with biallelic DADA2-associated mutations [7, 56]. Moreover, Trotta et al. reported on the presence of T cell large granular lymphocyte (T-LGL) phenotype in patients with DADA2. T-LGL leukemia is a rare chronic lymphoproliferative disease characterized by clonal rearrangement of the β chain, more rarely of the γ-chain of cytotoxic T cells (CTL). In two patients, there was a clonal expansion as assessed by flow cytometry analysis of the Vbeta repertoire. However, the patients also showed somatic gain-of-function mutations in *STAT3* in the CD8 T cell fraction, a known association with T-LGL, although in the typical cases, *STAT3* gain-of-function mutations are only present in the T-LGL clones [57].

Autoimmune features and positive autoantibodies, specifically, transiently positive lupus anticoagulant was present in 10% of patients. A systemic lupus (SLE) phenotype was described in one patient [45, 65].

### Hematological Disease

In some patients, cytopenia is the first manifestation or an accompanying feature of the disease. PRCA was described initially in three patients by Hashem et al. and Ben-Ami et al. and further confirmed by additional reports [47, 54]. The severe type of anemia in DADA2 may resemble Diamond-Blackfan anemia (DBA). It is unclear at this stage if these hematopoietic features are related to true bone marrow aplasia or are related to autoimmunity. Indeed, autoimmune hemolytic anemia and thrombocytopenia have been found in nine patients [1, 7, 57, 69]. Severe neutropenia is another important finding, and it has been reported in up to 10% of patients [7, 37, 55, 57–59, 71]. One patient presented with features suggestive of MonoMac syndrome due to GATA2 deficiency but was subsequently molecularly diagnosed with DADA2 [70]. Bone marrow biopsies showed reticular fibrosis and characteristic lymphoid aggregates [1, 47, 57, 58]. DADA2 should therefore also be considered in the differential diagnosis of bone marrow failure and idiopathic aplastic anemia, even in adults and in the absence of an inflammatory phenotype.

## Treatment

In the past, immunosuppressive therapies were used to control the severe systemic inflammation in patients with early-onset PAN and recurrent strokes. Steroids were the mainstay of treatment and have shown variable successes, however often with flares of inflammation and vasculitis upon tapering. Azathioprine, cyclosporine, tacrolimus, cyclophosphamide, and methotrexate have all been used yet with little success [1, 2]. One paper reported a patient with PAN who is a carrier for Familial Mediterranean Fever-associated mutation, which is common in the Turkish population, and a good response to colchicine [44]. Anti-interleukin-1 therapy with anakinra or canakinumab resulted in failure to control inflammation [1, 9]. Anti-IL-6 therapy with tocilizumab was successful in controlling the inflammation in a DADA2 patient with Castleman-like presentation, although a recurrent stroke was reported in other DADA2 patients treated with tocilizumab [40, 49]. Other case reports disclosed partially successful therapies with immunosuppressant drugs sirolimus and mycophenolate mofetil. At present, the mainstay of treatment consists of anti-TNF-agents (etanercept, infliximab, adalimumab). Consistent with this observation, Caorsi et al. reported that treatment with thalidomide gave better results than treatment with immunosuppressants with a complete response achieved in six patients. The drug was later discontinued due to neurological toxicity [50]. The treatment with biologic TNF-inhibitors was successful in controlling the fever episodes, vasculopathy, and prevention of strokes, in all patients reported. Unlike in standard care for stroke patients, it is recommended to discontinue treatment with acetyl salicylic acid and other anticoagulants as hemorrhagic stroke is a potential complication [1]. As for the reversal of cytopenia and immunodeficiency, the results are less clear, with reported failures [55] and opportunistic infections [55, 59]. The use of rituximab has been reported with variable success for controlling cytopenia (either ITP or neutropenia). In patients with documented hypogammaglobulinemia and a clinical immunodeficiency, immunoglobulin substitution, antibiotic, and antiviral treatment have been used routinely [7, 45].

As ADA2 is found in plasma, infusions of fresh frozen plasma were considered for substituting the ADA2 activity. However, the half-life of ADA2 and the large volumes of FFP make this approach less feasible [72].

As a more definitive treatment, HSCT has been reported to control both the immunological, the hematological, and the vascular phenotype of DADA2. Initial case reports of successful transplants in patients who presented with severe anemia have been recently extended to experience in 14 patients with DADA2 [7, 54, 55, 70, 71]. All patients who had HSCT were cured, and all are doing well at a median follow-up of 18 months. The longest follow-up was 10 years [55]. Of note, most patients were young. Age may be an important factor when considering HSCT as for most PID HSCT is more hazardous in adult age. Concerns remain regarding the optimal conditionings that are realistic, as a strong myeloid engraftment is crucial for cure [73]. Nevertheless, both reduced intensity conditioning and myeloablative conditioning resulted in successful transplant outcome. Next, as autoimmune complications were reported in 4/14 patients, addition of rituximab to the preparative regimen has been suggested [55]. Moreover, viral reactivations were common and needed careful monitoring and preemptive treatment where possible. Given the disease relapse with decline in chimerism in one patient, the selection of a donor needs careful evaluation. Family members carrying a disease-causing mutation should be avoided if possible [73]. Moreover, the decision to proceed to HSCT should be guided by the severity of the phenotype and the lack of response to treatment with TNF-inhibitors.

## Discussion

Deficiency of ADA2 was discovered by unbiased genetic studies in patients with early-onset vasculopathy/vasculitis who were suspected to have a monogenic disease. Although the protein assay for the ADA2 activity has been used to assess disease activity in many inflammatory conditions, ADA2 was never considered a candidate gene for any immune disorder. This is likely due to the fact that the function of ADA2 has been poorly characterized, owing to the lack of mouse ortholog. Identification of patients with the ADA2 deficiency has now raised a new interest in studying the role of ADA2 in the regulation of immune signaling.

DADA2 can manifest with a broad spectrum of features including vasculitis/vasculopathy, livedo reticularis/cPAN, lacunar strokes, and systemic vasculitis involving all organs, esp. the liver. More recent reports have stressed hematological manifestations with PRCA, thrombocytopenia, and neutropenia. Also, immunodeficiency is a non-negligible manifestation of the condition with patients presenting a CVID phenotype, yet also a striking susceptibility to herpes virus infections and lymphoproliferation. Surprisingly, these immunodeficiency and hematological manifestations can be present in the absence of fever and vasculitis. Due to this highly variable phenotype, patients with ADA2 may not present to the clinical rheumatologist but also to the hematologist, the immunologist, and the hepatologist. Given the important morbidity and mortality, a high index of suspicion is needed for early diagnosis and intervention. The mainstay of treatment is TNF-inhibition, which is successful in suppressing inflammation and in prevention of vascular events. HSCT may be an option in patients presenting with hematological disease and immunodeficiency not responding to TNF-inhibitors. Future therapies may include recombinant ADA2 protein or gene therapy. In order to develop directed therapies, further studies on the function of ADA2 and the pathophysiology of ADA2 deficiency are necessary. This will ultimately not only aid in optimizing the treatment of patients suffering from this devastating condition, but ADA2 might also be a new target for antiangiogenic therapy in certain types of human cancer [64].
